# Viral inactivation by light

**DOI:** 10.1186/s43593-022-00029-9

**Published:** 2022-09-26

**Authors:** Mohammad Sadraeian, Le Zhang, Farzaneh Aavani, Esmaeil Biazar, Dayong Jin

**Affiliations:** 1 Present Address: Institute for Biomedical Materials and Devices (IBMD), Faculty of Science, University of Technology Sydney, Sydney, NSW 2007 Australia; 2 Department of Oral and Maxillofacial Surgery, Division of Regenerative Orofacial Medicine, University Hospital Hamburg-Eppendorf, 20251 Hamburg, Germany; 3 Department of Biomedical Engineering, Islamic Azad University, Tonekabon Branch, Tonekabon, Iran; 4 UTS-SUStech Joint Research Centre for Biomedical Materials & Devices, Department of Biomedical Engineering, College of Engineering, Southern University of Science and Technology, Shenzhen, Guangdong China

**Keywords:** Antiviral therapy, Enveloped virus, Photo-inactivation, Ionizing radiation, Nuclear radiations

## Abstract

Nowadays, viral infections are one of the greatest challenges for medical sciences and human society. While antiviral compounds and chemical inactivation remain inadequate, physical approaches based on irradiation provide new potentials for prevention and treatment of viral infections, without the risk of drug resistance and other unwanted side effects. Light across the electromagnetic spectrum can inactivate the virions using ionizing and non-ionizing radiations. This review highlights the anti-viral utility of radiant methods from the aspects of ionizing radiation, including high energy ultraviolet, gamma ray, X-ray, and neutron, and non-ionizing photo-inactivation, including lasers and blue light.

## Highlights

- Inactivation of enveloped viruses has been explored in several ways to prevent the widespread development of their pathogenicity, but the ideal method has yet to be established.
- Understanding the physicochemical mechanisms of viral inactivation using irradiation methods will help to design and engineer tools for viral elimination with enhanced efficacy, safety and long-term use.
- An overview of the mechanism of the following physical waves on the inactivation of enveloped viruses, based on lipid, protein and genome damages, will be discussed.
- This article focuses on the light-based strategies for virus inactivation using gamma rays, X-rays, neutron rays, UV rays, low-energy electron irradiation (LEEI), blue light, and laser-based photoinactivation.

## Introduction

Historically, viruses have been the causative agent of the most significant human diseases such as human immunodeficiency virus (HIV), various types of influenza viruses [1] and SARS-CoV-2 [2]. As one of the most devastating pandemics in human history, the “1918 influenza pandemic”, caused by an H1N1 virus, was responsible for tens of millions of casualties in the early twentieth century [1]. For several hundreds of years, light has been recognized as a potential antimicrobial device to fight against infection diseases. Niels Ryberg Finsen made numerous efforts to use concentrated light rays to treat lupus vulgaris, the most common Mycobacterium tuberculosis skin infection, which earned him the Nobel Prize in Physiology/Medicine in 1903 [3, 4]. The exposure to the healing rays has become a common form of light therapy against bacterial infections, as well as wound infections, psoriasis, acne vulgaris, rickettsia, depression, jaundice, and a host of other diseases. To date, studies continue to explore the application of rays, from sunlight to non-invasive laser light, against infective pathogens [3, 4].

In general, the shapes of viruses include filamentous, icosahedral, enveloped, and head-and-tail. Animal viruses, such as coronaviruses, are often enveloped and have a punched-in spherical appearance. Enveloped viruses are surrounded by an outer lipid membrane that makes them sensitive to some environmental changes, therefore it is easier to inactivate these viruses compared to non-enveloped viruses. Membrane of enveloped viruses mainly consist of three types of structural proteins; spike protein (S), membrane protein (M), envelope protein (E). The internal parts of viruses include a nucleocapsid protein (N). Many viruses, whether enveloped or non-enveloped, carry stealthy spikes that bind to host cell receptors [5]. The binding of spike protein to the receptor provokes fusion of the viral envelope with the cell membrane, resulting in internalization of the viral nucleocapsid into the cytoplasm [6, 7]. In many cases, the spike protein penetrates the plasma membrane of infected cells and fuses adjacent cells to form syncytia [8].

A variety of drugs have been synthesized for intervening virus activities by interrupting and blocking virus membranes, inactivating genetic contains, and boosting the immune system. Some drugs and active molecules are able to inactive virus or cell receptors to inhibit their attachment [9, 10]. Antiviral drugs have major limitations. Inactivation of viruses by chemical drugs requires passing across the rigid capsid of the virus membrane. Chemotherapies (drugs) can interfere with the virus life-cycle after infection, but the host may suffer harmful side effects [11]. Besides, rapid viral mutations, alteration of the spike protein, and subsequently changing the viral antigenicity are considered critical challenges to vaccine and drug designation, which reduce their effectiveness [12]. Many pathogenetic viruses, including the novel coronavirus, can alter their genetic code to survive against effective drugs [13]. With the example of AZT drug resistance in HIV treatment leading to a healthcare cost crisis, the search for alternative therapies that can attack multiple targets in the virus is becoming more urgent to prevent viral resistance. Apart from drug resistance, common treatments should be used for several weeks to be effective against microorganisms [14].

**Fig. 1:**
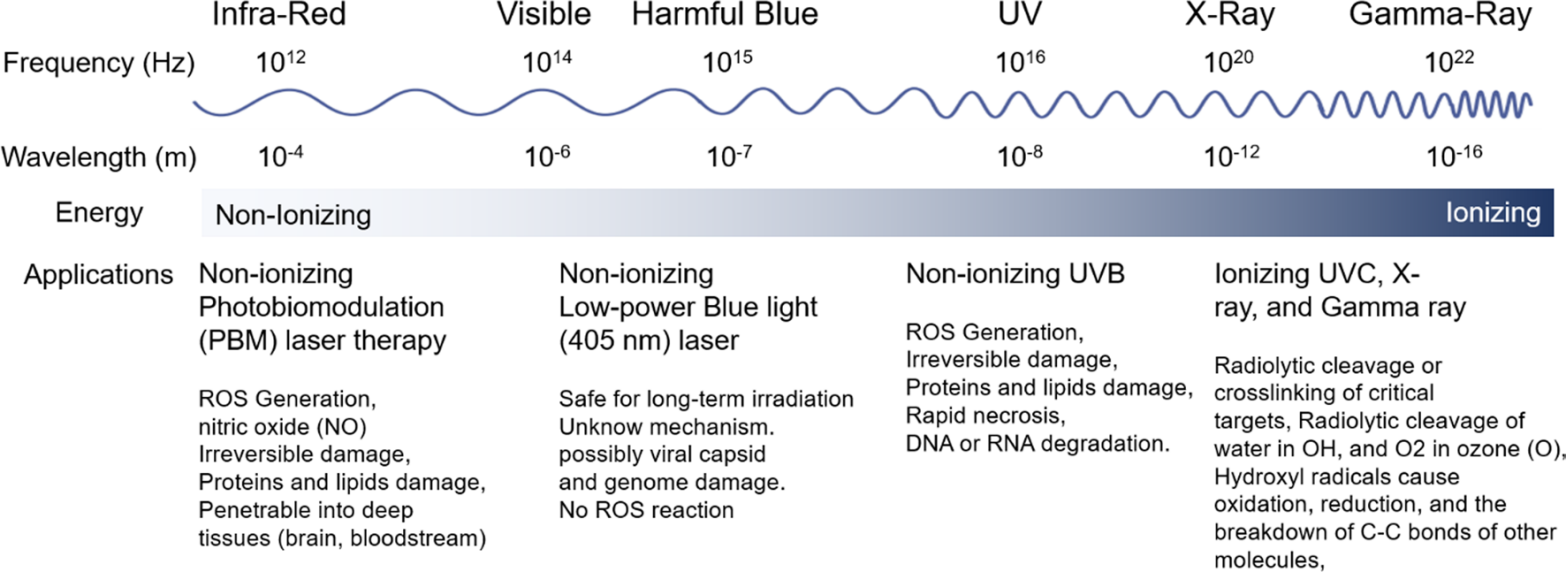
The virus inactivation using electromagnetic waves. Electromagnetic waves from the range of infra-red waves to gamma due to different physical functions and their effects on different segments of viruses have caused the virus to be inactivated

As alternative antiviral therapies, scientists have introduced physical methods and techniques to disrupt viral functionalities. For example, it has been demonstrated that the application of light beams can be useful for viral inactivation (Fig. 1) [15]. A combination of physical methods and an understanding of the physicochemical mechanisms of viral inactivation will be effective in designing and developing tools for viral elimination with improved efficacy, safety, and long-term application [16]. This article reviews irradiation-dependent physical methods and studies related to the inactivation of viruses, especially enveloped viruses such as HIV, influenza, or hepatitis, which are responsible for serious problems in humans (Table 1).

**Table 1:** Studied different techniques for the inactivation of viruses

Techniques	Light radiation	Viruses	Killing dose	Refs
Ionizing radiations	Far-UVC (222 nm), 0.56 mJ/cm^2^, 3–25 min	Human Coronavirus HCoV-229E	D 99.9 (mJ/cm^2^): 1.7, 25 min	[17]
	Far-UVC (222 nm), 0.39 mJ/cm^2^, 3–25 min	Human Coronavirus HCoV-OC43	D 99.9 (mJ/cm^2^): 1.2, 25 min	[17]
	UVC (254 nm), 10, 60, 360 mJ/cm^2^, 1, 6, 36 s	SARS-CoV-2 pseudovirus	D 99.99 (mJ/cm^2^): 36 s	[18]
	UVC (254 nm), 120 mJ/cm^2^ for 30 min	Murine coronavirus mouse hepatitis virus (MHV)	Virus titer (PFU/mL): 2 log reduction at 2 h and 1 log reduction at 8 h	[19, 20]
	UVC (260 nm)	Severe acute respiratory syndrome coronavirus (SARS-CoV)	Cytopathic effect (CPE): Without detectable CPE after 60 min radiation	[21]
	Deep UV light-emitting diode (DUV-LED) (280 ± 5 nm)	SARS-CoV-2	D 99.9: 75 mJ/cm^2^, 10 s	[22]
	Gamma ray, 10 kGy	Influenza A	–	[23]
	Gamma ray, 0–50 kGy	Polio Virus 1 and 2 Sabin strains	CCID_50_: 0 at 40 kGy for PV1-S and 45 kGy for PV2-S	[24]
	Neutrons and gamma photons, 0–15.6 kGy	Influenza A X31/H3N2 and PR8/H1N1	Gamma: 2–threefold more effective than n with (RBE) range of 0.43–0.65 TCID_50_: 7.08 and 15.07 with gamma and neutron radiation (X31) TCID_50_: 5.77 and 14.21 with gamma and neutron radiation (PR8)	[25]
	Electron beam (LEEI).200 keV, 30 kGy	Influenza A (H3N8), Porcine reproductive and respiratory syndrome virus (PRRSV), Equine herpesvirus 1 (EHV-1),	Log TCID_50_/mL: 0 for Influenza A (H3N8) at E-Beam absorbed Dose: 29.9 kGy and for PRRSV and EHV-1: 10.4 kGy	[26]
	X-ray, 220 keV, 17.5 mA with 0.2 mm Al filtration	Zika virus (ZIKV) African strain MP1751, RVFV-X	1-log_10_ decimal-reduction value (D_10_): 12.8 kGy for ZIKV-X and 15.79 kGy for RVFV-X	[27]
Non-ionizing radiations	Blue light (455 nm), 50 mW/cm^2^, 40 h (= 7200 J/cm^2^)	Bacteriophage phi6	Virus titer (PFU/mL): 1 log reduction dose 2130 J/cm^2^	[28]
	Blue light (405 nm), 78.6 mW/cm^2^, 4.5 h (= 1272 J/cm^2^)	Bacteriophage phi6	Virus titer (PFU/mL): 1 log reduction dose 430 J/cm^2^	[29]
	Blue light (405 nm), 0.035 mW/cm^2^ and 0.6 mW/cm^2^ for SARS-CoV-2 and IAV, respectively, for 4 h	SARS-CoV-2 and Influenza A virus (IAV)	Virus titer (PFU/mL): 0.3288 log10 and 0.5609 log10 reduction for SARS-CoV-2 and IAV, respectively	[30]
	Blue laser, Low-power ultrashort pulse (USP) (425 nm). 3.4 J/cm^2^	M13 bacteriophage, Murine cytomegalovirus	Log (pfu): 5 for M13 bacteriophage at 1 h of laser exposure time	[31]
	Blue Femtosecond laser (400 nm). 20 mJ/pulse	M13 bacteriophages	Log 10 (Loud reduction): 5.8 ± 0.3 and 2.9 ± 0.15 at 15 and 2 min, respectively	[32]
	Blue laser (408 nm), Low-power ultrashort pulse (USP), 150 mW	Murine Norovirus	> 3 log10 inactivation at 3 h	[33]
	Red Laser, LLLT (660 nm), 2–10 J/cm^2^, 626 s	HIV-1	Luciferase activity: Reduction in infected cells (8 J/cm^2^) and no inhibitory effect in uninfected cells	[34]
	Infrared Femtosecond laser (800 nm), 4 μW, 10 ms	HIV-1	Luciferase activity: Two-fold reduction with Radiation and Raltegravir for 30 min	[35]

## Ionizing radiations

Ionizing radiation can inactivate pathogens by destroying the genome but causing less damage to structural components, such as the protein membrane. Researchers use ionizing radiation to produce various types of vaccines and in sterilization processes [36, 37]. Although this method is suitable for the sterilization of biological samples or producing inactivated vaccines, it is limited to the environment and personal protective equipment (PPE). Moreover, ionizing radiation such as X-ray and Gamma ray have been widely used in medical sciences, such as radiology in diagnosis of diseases and treatment. Energetic photons, including gamma rays, X-rays, and UVC light, can release their energy when interacting with coronaviruses and damage the viral genome either directly by radiolytic cleavage of genetic material or indirectly by the action of radicals on viral nucleic acids, as shown in Fig. 1 [38, 39]. Therefore, certain intensities of X-ray, gamma-ray, and UVC light could be used to noninvasively inactivate SARS-CoV-2 virus replication and proliferation in the lung of COVID-19 patients.

### UV

#### UV classification based on wavelength

Ultraviolet (UV) radiation is the region of non-ionizing radiation that is adjacent to ionizing radiation in the electromagnetic spectrum. Therefore, low-energy UV irradiation is considered non-ionizing radiation, while high-energy UV irradiation is a form of ionizing radiation, because the high energy is sufficient to ionize an atom or molecule by stripping electrons from it [40]. UV radiation is a well-known means of inactivating all known microorganisms and viruses. However, on the downside, UV radiation plays a key role in the initiation phase of skin cancer by causing DNA damage [41]. Skin cancer has stochastic effects, and no threshold dose can be defined to determine the effects or symptoms. In general, the long-term effects are worse with continuous exposure, and some precautions must be taken to minimize the risk.

The effectiveness of UV as a disinfectant is highly dependent on the wavelength of the incident photons, including UVA (320–400 nm), UVB (280–320 nm), and UVC (200–280 nm). UVC is the strongest UV light with the most potent antimicrobial/antiviral properties. UVA (320–400 nm) is less energetic but 20 times more intense and therefore induces the formation of cyclobutene pyrimidine dimers (CPDs) along with a variety of oxidatively generated lesions, such as single strand breaks and oxidized bases. UVB, 280–320 nm leads to the formation of CPDs and pyrimidine (6–4) pyrimidone photoproducts (64PPs) [42]. UVC (200–280 nm) causes several types of damage to DNA or RNA, including photochemical changes, cross-linking, and oxidative damage [42].

#### Effects of UVC on virus components

**Fig. 2:**
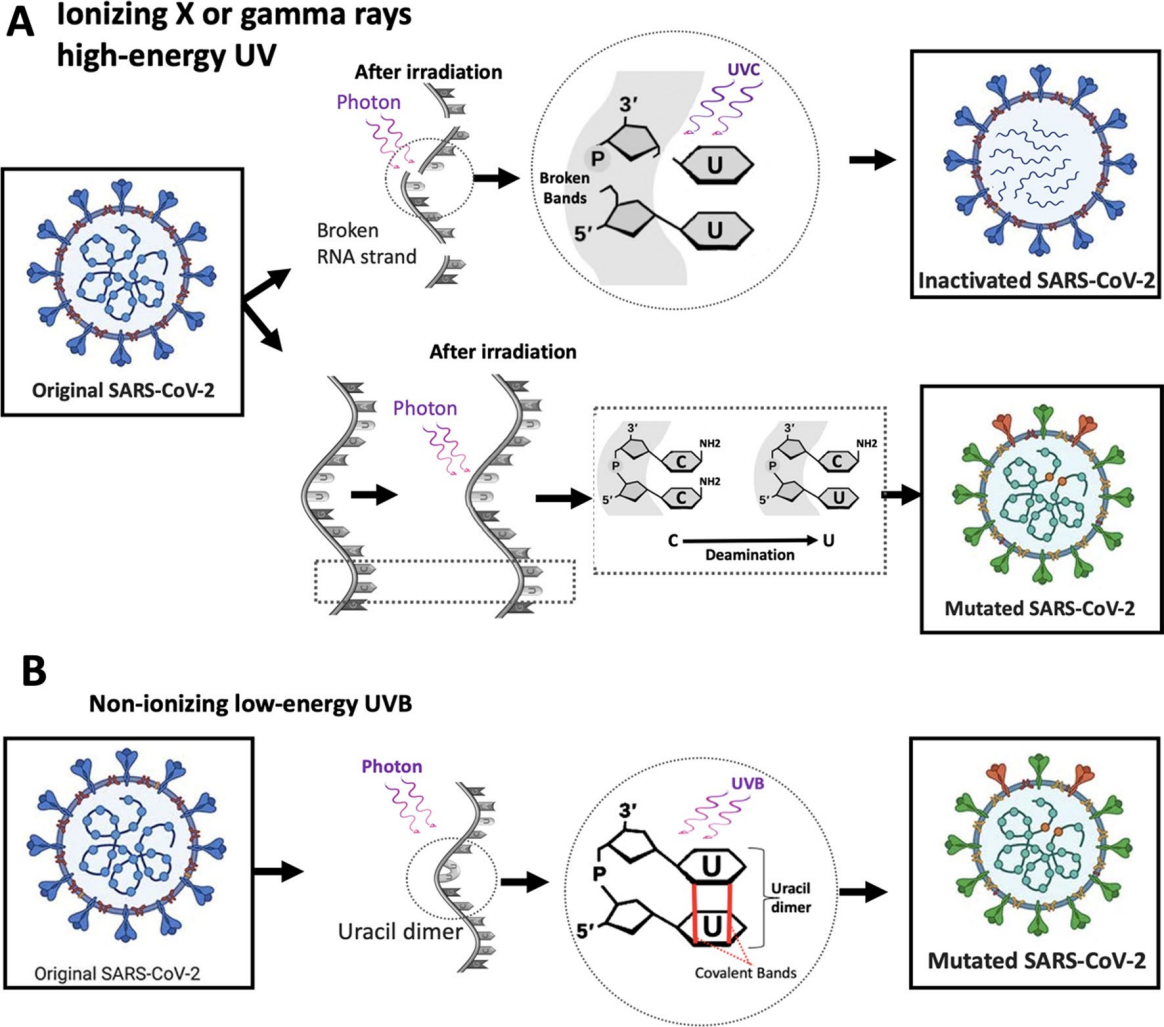
Exposure to either ionizing (**A**) or nonionizing (**B**) radiation can each induce mutations in RNA, although by different mechanisms

The adverse effects of UVC depend on the light intensity, wavelength, source-target distance, beam width, and also the target (e.g., virus species in terms of morphology and genome) [42]. UVC damages the viral genome, but there are some reports of damage to viral proteins. In the case of DNA viruses, two major UV-induced lesions, pyrimidine dimers (Py-Py) and pyrimidine (6–4) pyrimidones [Py(6–4)Py], which arise at the same nucleotide sites, are lethal and mutagenic [43, 44]. In contrast, RNA differs from DNA mainly in the presence of uracil nucleotides instead of thymine. Therefore, UV irradiation give rise to several RNA photoproducts, mainly from adjacent pyrimidine nucleotides, such as uracil dimers, as well as RNA–protein cross-links [45]. These lesions inhibit RNA synthesis primarily through the formation of 6–4 photoproducts [45, 46], also block the reverse transcriptase enzyme in the transcription of cDNA strands [47]. Although the propagation mechanism of SARS-CoV-2 is independent of reverse RNA transcription and genome integration, UV-induced lesions can still lead to the formation of uracil dimers, capsid instability and degradation, and covalent binding of the protein to viral RNA (Fig. 2) [48, 49].

In addition to damaging the viral genome, there are some reports on the effect of UVC on viral protein depending on the virus type. For example, UVC irradiation causes oxidative damage to the viral capsid protein in non-enveloped viruses, such as feline calicivirus [50] and bacterial phage MS2 [51]. Another study showed that UVC alone or with titanium dioxide TiO_2_ photocatalysis (UVC-TP) destroyed murine norovirus 1 (MNV-1) viral capsid protein in addition to viral genomic RNA [52]. Furthermore, cross-linked RNA–protein complexes were observed in UVC-irradiated polioviruses [53]. Therefore, UVC-induced damage varies among different virus types in terms of genome or protein damage. Consequently, the mechanism of UVC inactivation needs to be tested in different viruses. All conventional UV lamps usually use mercury discharge lamps, especially low-pressure mercury vapor lamps, with a strong emission peak at 254 nm, which is close to the RNA absorption maximum at about 260 nm.

#### Effects of UVC on SARS-CoV-2

Although UV-based viral disinfection is generally considered safer than chemical disinfection, mercury vapor lamps are a public health concern. As an alternative, UV LEDs are not only safer but also more compact, low-cost and long-lasting [39]. Lytle and Sagripanti reported that 254 nm UV at 3.1 J/m^2^ can reduce viable coronaviridae viruses to 37% (D37) [54]. In addition, some studies have shown that UVC can inactivate coronaviruses, including SARS-CoV and Middle East Respiratory Syndrome Coronavirus (MERS-CoV), within 5 min of exposure time at a distance of 4 feet between source and target [55].

Since the onset of the COVID -19 pandemic, several reports have highlighted the importance of UVC as the most useful method for rapidly reducing the viability and infectivity of SARS-CoV-2. Sabino et al. [56], developed a controlled assay to study the UVC inactivation kinetics of SARS-CoV-2 and determined the lethal UVC doses in vitro. For this purpose, a low-pressure mercury lamp emitting UVC radiation at 254 nm was used. An aliquot of the SARS-CoV-2 stock was thawed and 100 µL was diluted in 900 µL of high glucose DMEM without addition. Then, 200 µL of this dilution was added to the wells and irradiated with the lamp placed 30 cm above the plate to allow uniform irradiation across the wells of the plate (2.2 ± 0.2 mW/cm^2^). Light was delivered for 2 s, 30 s, and 120 s, corresponding to doses of 4.4, 66, and 264 mJ/cm^2^, respectively. The samples were then used to inoculate Vero cell cultures, and viral RNA was quantified using RT-qPCR technique. LD90 was achieved at a very short irradiation of 0.01 s (0.016 mJ/cm^2^).

Inagaki et al. [22] tested the potential virucidal activity of UV-LED with a wavelength of 280 nm ± 5 nm, against SARS-CoV-2. To evaluate UV-LED inactivation, aliquots of 150 µL of the virus broth (3.7 × 10^4^ PFU/mL) were placed in the center of a 60-mm Petri dish and irradiated with 3.75 mW/cm^2^ at a working distance of 20 mm for a series of times (n = 3 each time for 1 s, 10 s, 20 s, 30 s, and 60 s, and each dose corresponds to 3.75, 37.5, 75, 112.5, and 225 mJ/cm^2^). The reduction in infectious titer was 87.4% for 1 s of irradiation, and the rate increased to 99.9% for 10 s of irradiation.

In a similar study by Biasin et al. [57], the antiviral efficacy of UVC irradiation on SARS-CoV-2 was investigated experimentally for a range of radiation doses and virus concentrations (1000, 5, 0.05 MOI). The results showed that a UVC dose of 3.7 mJ/cm^2^ was sufficient to achieve inactivation of more than 3log, with no evidence of viral replication. Furthermore, complete inactivation was observed at 16.9 mJ/cm^2^, at all viral concentrations. These results are important for the development of UVC-based inactivation methods to combat SARS-CoV-2 infection [54, 55, 58]. Short-wave UVC (200–280 nm) is the most harmful type of UV radiation.

A pseudotyped model can be used in place of a real virus for the radiation-based strategies to understand the mechanism of viral photoinactivation without having to work in BSL-3. Sadraeian el al. [18] examined the titer of SARS-CoV-2 Spike pseudotyped model after UV irradiation at 254 nm using Luciferase and qPCR molecular assay. In this study, spike pseudotyped lentivirus was used instead of actual (authentic) virus. This is because, unlike the actual RNA virus, whose viral replication is independent of the host genome, the pseudotyped model allowed not only the study of RNA viral load but also DNA integration into the host genome. Furthermore, the generation of pseudovirus without spike could be used as a negative control, helping to compare the viral particles in two forms of enveloped and non-enveloped (naked) viruses, which is important for side-by-side comparison.

#### Deep UV-based LEDs

Deep UV-based LEDs with higher efficiency can simplify the introduction of portable disinfection systems, such as UVC-equipped masks, and reduce the energy consumption, leading to faster and easier technological implementation of new products [59]. Minamikawa et al. [60] optimized the irradiation device for deep UV LEDs by quantitative assessments of DUV-LED irradiation effects on SARS-CoV-2. They also, determined the culture media suitable for inactivation of SARS-CoV-2 and developed an irradiation system using commercially available DUV LEDs operating at a central wavelength of 265 nm, 280 nm, or 300 nm. At all wavelengths, the number of plaques decreased exponentially with the total dose of DUV-LED energy. The DUV-LED, operating at a wavelength of 265 nm, showed the highest inactivation effect on SARS-CoV-2. To achieve 99.9% inactivation of SARS-CoV-2, the total energy dose required by DUV-LED was found to be 1.8 mJ/cm^2^ for 265 nm, 3.0 mJ/cm^2^ for 280 nm, and 23 mJ/cm^2^ for 300 nm. Using these quantitative data, the effectiveness of inactivating SARS-CoV-2 can be estimated based on the transmission levels in each environment. To minimize DUV-LED light absorption by the culture media during irradiation, they reported that the culture media suitable for DUV-LED irradiation should contain a minimum of EMEM media and FBS additive to significantly reduce light absorption.

#### UV signature mutations

Both ionizing and non-ionizing radiation can cause mutations in DNA, albeit through different mechanisms. Strong ionizing radiation such as high-energy UVC, X-rays, and gamma rays can cause single- and double-strand breaks in the DNA backbone through the formation of hydroxyl radicals upon irradiation. Methods based on UV irradiation can promote genetic mutations and create new variants that are potentially more stable and transmissible or move toward eradication. These mutations forced by irradiation are known as "mutational signatures", which could be tracked by high-throughput nucleotide sequencing [61]. Ionizing radiation can also change bases; for example, cytosine can change to uracil by deamination (Fig. 2).

Non-ionizing radiation can induce the formation of dimers between two adjacent pyrimidine bases. In the case of DNA viruses, this dimerization could occur between two thymines [62], within a nucleotide strand, whereas uracil dimerization can occur in RNA viruses [63]. The formation of uracil/thymine dimers can lead to frameshift or point mutations in the genome known as mutational signatures [64]. While previous studies classified mutations based on energy and wavelength, more recent studies have shown that both high- and low-energy radiation can lead to mutational signatures and that UV signature mutations induced by UVC, UVB, and UVA are generally similar [65]. Analysis of SARS-CoV-2 genome sequences from around the world has shown that the rate of UV mutational signatures is higher than expectation if these events occurred randomly [63]. The global mutation spectrum of SARS-CoV-2 overwhelmingly indicates C→U and G→U substitutions. Incidentally, several UV irradiation techniques are currently being used to reduce the risk of viral infection, which may accelerate the emergence of C→U mutations in global SARS-CoV-2 genomes. The prevalence of UV mutational signatures in SARS-CoV-2 beyond the general replication error-induced mechanism of mutagenesis necessitates that we vigilantly monitor the long-term implications of irradiation-mediated viral inactivation strategies. Because these strategies can reduce transmission of viral infections, we need to closely monitor the occurrence of UV mutational signatures in global SARS-CoV-2 genomes.

### Nuclear radiations

#### Gamma ray

Inactivation of viral pathogens by Gamma radiation can be used in sterilization and the production of biological reagents, including the production of noninfectious viral antigens. Chemical inactivation by formaldehyde or β-propiolactone has traditionally been used to render viruses harmless for vaccine production [66]. However, the process of chemical inactivation is lengthy and requires many additional purification steps. During the purification process, product mass may decrease, viral proteins may be degraded, and toxicity may occur. In contrast, gamma irradiation of the SARS-CoV-2 avoids unwanted toxicity while maintaining efficacy, as demonstrated in several animal models. By preventing viral shedding, gamma irradiation also becomes a more cost-effective method of virus purification. Leung et al. [66], investigated that a radiation dose of 1 mrad is required to completely inactivate 106.5 TCID50/mL of SARS-CoV-2. Gamma irradiation can be used as a reliable method to inactivate SARS-CoV-2 with minimal impact on the subsequent PCR assay. The effect of gamma irradiation on PCR sensitivity is dose-dependent up to 0.5 mrad, after which there was no further reduction. Turan et al. [67], demonstrated that OZG-38.61.3 vaccine candidates prepared with gamma-irradiated inactivated SARS-CoV-2 virus produced neutralizing antibodies that were particularly effective in the 10^14^ viral RNA copy formulation and effectively protected transgenic human ACE2-expressing mice from SARS-CoV-2 virus. The efficacy and safety dose (either 10^13^ or 10^14^ viral RNA copies per dose) of OZG-38.61.3 were found to be safe in BALB/c and transgenic mice.

Studies suggest that the genetic material, rather than the protein and lipid envelopes, is likely to be the primary target for viral inactivation [68]. Nims et al. [69] reported that the presence or absence of a viral envelope does not appear to be a major factor for inactivation by γ-irradiation. Single-strand breaks (for single-stranded viruses) and double-strand breaks (for double-stranded viruses) are generally sufficient to inactivate the viral genome [70]. Feldmann and colleagues showed that inactivation is inversely correlated with genome size [71].They measured radiation doses for a 6 log10 reduction and found 2 mrads for coronaviruses (∼29 kb genome size), 4 mrads for filoviruses (∼19 kb), 8 mrads for arenaviruses, bunyaviruses, orthomyxoviruses, and paramyxoviruses (∼13 kb), and 10 mrads for flaviviruses (∼9 kb). Viruses with single-stranded nucleic acid exhibit the highest radiosensitivity. Hume and colleagues [68] reported that the three enveloped single-stranded RNA viruses of similar size, namely morbillivirus (90–150 nm), bunyavirus (90–120 nm), and rhabdovirus (70–150 nm), had comparable D 10 values (2.53, 2.61, and 2.71 kGy, respectively) when irradiated with the same experimental protocol. Leung et al. cultured SARS-CoV-2 on Vero cells in the presence of 1% fetal bovine serum and 1% l-glutamine. The virus-containing supernatants were titrated after irradiation [66]. The D10 was 1.6 kGy and complete inactivation of SARS-CoV-2 was achieved with an absorbed dose of 10 kGy, a value lower than the 20 kGy previously reported for the similar SARS-CoV [71].

Inactivation by gamma radiation has been proposed as an alternative method for inactivating viral replication by damaging nucleic acid while retaining immunogenicity. Mullbacher et al., [72] demonstrated a high cross-protective immune response of the irradiated influenza A virus against other influenza A strains. In compare to UV-irradiation, the gamma-irradiated influenza vaccine elicited greater T-cell cross-reactivity and protected mice against a heterologous influenza virus. Another study [23, 72] showed that a single dose of non-adjuvanted intranasal gamma-irradiated influenza A vaccine (GammaFlu) provided significant protection in mice, mediated mainly by cytotoxic T cells. Unlike the chemical inactivation method, gamma irradiation preserved the functional domains of viral proteins, which facilitated uptake and presentation on the primary histocompatibility complex class I (MHC-I) of antigen-presenting cells (APCs). For small viruses (core size ≤ 20 nm) such as Picornaviridae (genome size: 7 kb) and Retroviridae (genome size 7–10 kb), high D10 levels were detected in the order of 0.55 and 0.35 kGy, respectively [73].

In contrast, large viruses (core size 75–150 nm), such as Bunyaviruses (genome size 10–22 kb) and Paramyxoviridae (genome size 15 kb), have lower D10 values on the order of 0.2 kGy. Thus, it is possible to determine the radiation dose required to achieve a sterility assurance level (SAL) for any virus preparation by determining the initial virus titer and D10 value. Influenza virus is a relatively large virus with a core size of 80–120 nm and a genome size of 12 kb, consisting of at least eight individual negatively stranded RNA fragments. The International Atomic Energy Agency's Manual for Radiation Sterilization of Medical and Biological Materials states that irradiation with 0.65 kGy of γ-rays results in complete loss of infectivity of influenza virus, but destruction of hemagglutinating activity requires irradiation with more than 200 kGy [74]. Influenza vaccine preparations inactivated with gamma rays have been formulated with a radiation dose of 10 kGy. These preparations have been shown to induce cross-protective immunity against heterotypic viral challenges in mice.

In addition, sterile H5N1 avian influenza virus generated by irradiation at 50 kGy was used to produce neutralizing monoclonal antibodies for diagnostic purposes and for possible future therapeutic use [75]. This approach has been tested in preclinical studies by Gamma Vaccines Pty (Manuka, A.C.T., Australia) and is now being tested in a full clinical trial. The most promising result was obtained with the gamma-irradiated, completely killed attenuated HIV vaccines SAV0001 and Remune [76]. This vaccine is derived from a recombinant clade between subtypes. An envelope and clade G Cag are inactivated by sequential application of beta-propiolactone (BPL) and gamma irradiation. The HIV envelope glycoprotein gp120 is depleted during preparation and inactivation [77]. Although this was a large-scale multicenter phase III HIV trial, there were no significant differences in the incidence of opportunistic infections (OIs) or death in patients receiving antiretroviral therapy. HIV-related OIs include pneumonia, Salmonella infection, candidiasis, toxoplasmosis, and tuberculosis (TB). For people with HIV, the primary protection against OIs and death-related OIs is to take HIV antiviral drugs. In addition, in subjects treated with Remune, a statistically significant decrease in viral load led to an increase in CD4+ T cell count and an enhancement of HIV-1-specific antibody responses. Researchers developed SAV001 at Western University, Canada [78]. This was one of the clinical trials on preventive HIV vaccine based on a genetically modified virus. The nef and vpu genes were removed from the HIV-1 strain to produce the attenuated strain. The env signal peptide was replaced with the honeybee antimicrobial peptide melittin to increase virus replication and production. Thus, this genetically modified HIV-1 strain is nonpathogenic and can be generated in large quantities in a cell culture-based system. This modified virus was produced as a killed vaccine by harvesting HIV-1 that was completely inactivated by sequential gamma irradiation and aldrithiol-2 [79].

Gamma ray can cause direct and indirect damage to proteins, lipids, and nucleic acids and subsequently lead to loss of cellular viability or viral activity. The likelihood that a small amount of ionizing radiation will cause lethal damage is usually directly related to genome size. Due to differences in genome size, higher radiation doses (e.g., 20–45 kGy) are usually required to inactivate viruses compared to bacteria or mammalian cells [80, 81]. Tobin et al. [24] developed an ionizing radiation-inactivated Sabin-based vaccine using a reconstituted Mn-decapeptide (MDP) antioxidant complex derived from the radiation-resistant bacterium Deinococcus radiodurans. This extremophilic bacterium has Mn^2+^ peptide antioxidants that protect proteins from oxidative damage caused by extreme radiation exposure [24, 82]. They reported that MDP can protect immunogenic neutralizing epitopes in picornaviruses. MDP protects epitopes in Sabin strains of poliovirus 1 and 2 (PV1-S and PV2-S, respectively), but viral genomic RNA is not protected during supralethal irradiation. The results showed complete inactivation of PV by doses of 45 kGy, preserving the surface epitopes required for high immunogenicity during vaccination. Inactivated Sabin viruses stimulated equivalent or enhanced neutralizing antibody responses in Wistar rats compared with commercially used IPV products. Their approach reduces the biosafety risk of the current method of PV vaccine production by using the Sabin strains instead of the wild-type neurovirulent strains. In addition, the radiation inactivation approach may provide a simpler, faster, and less expensive method for producing a more immunogenic IPV. Gamma irradiation is a well-known method for inactivating viruses, and this vaccine approach could be adapted to any desired pathogen [24]. In addition, these treatments would be potentially safer for patients than many antiviral drugs, which can cause troublesome side effects.

#### Neutron-ray

Neutron beams are high-energy and are often used to sterilize viruses, as neutron radiation is about ten times more effective than gamma rays in inactivating viruses [83]. Neutron beams are high-speed nuclear particles (hundreds or even thousands of meters per second) that have an extraordinary ability to penetrate materials. Neutron sources are typically radioisotopic neutron sources and monoenergetic neutron generators. The typical radioisotopic neutron sources used in nuclear technology are ^252^Cf (Californium-252) and ^241^Am-Be (Americium-241/Beryllium). The half-life of the ^252^Cf neutron source is about 2.6 years, while the half-life of ^241^Am-Be is 432.2 years. A closed SARS-CoV-2 inactivation vessel 82 was created using the Monte Carlo method [83], researchers used a 13 MeV neutron radiation with an intensity of 10^12^ n/s and the sample was 5 cm away from the source.

In another study, ionizing radiation was used to inactivate some of the water-born bacterial virus (T2 phage) via the formation of H and OH radicals and more long-lived products such as H_2_O_2_ and HO_2_ in the presence of dissolved oxygen. The fast electron radicals can inactivate the virus according to two models, irradiation-dependent inactivation in the solution medium, which is time-limited, and prior irradiation of the medium followed by addition of the virus to medium [84]. Neutrons offer the in the possibility of identifying individual subunits by advanced mutation, which allows detection of specific regions (RNAs, proteins, and lipids) in complexes. When frozen influenza AX31/H3N2 and PR8/H1N1 samples were exposed to gamma and neutron doses ranging from 0 to 15.6 kGy, viral titers, determined by tissue culture infectious dose (TCID50) and plaque forming unit (PFU) assays, show that gamma rays were 2–3 times more effective than neutron rays, with a relative biological effectiveness (RBE) of 0.43–0.65. PAGE analysis of viral proteins and RNAs showed no macromolecular damage. The dependence of the magnitude of D10 on the titer test in this study suggests that more than one titer method should be used to determine whether complete inactivation has occurred [25].

### Electron radiation

#### Low-energy electron irradiation (LEEI)

Low-energy electron irradiation (LEEI) is being introduced as an alternative inactivation method for pathogens in liquid solutions. Like other types of ionizing radiation utilized for viral inactivation, LEEI mainly acts by destroying genome but causes less damage to structural components like protein. As the electrons have a limited penetration depth, LEEI is currently used for sterilization of surfaces. Inactivation of pathogens in liquids requires irradiation of the culture in a thin film to ensure complete penetration. Fertey and colleagues [85] have shown that LEEI effectively inactivates two single-stranded RNA viruses (influenza A (H3N8) and PRRSV), a double-stranded DNA virus (EHV-1), and a Gram-negative bacterium (*E. coli*). In this study, samples were irradiated with LEEI using a 200 keV electron beam at 4 °C. The LEEI-inactivated influenza A viruses induced protective immune responses in animals which was analyzed by virus neutralization assays and viral load determination. The LEEI dose, required to completely inactivate *E. coli*, was lower than that for the different viruses. For influenza A viruses, a dose of 9.6 kGy was previously reported for a 10,000-fold reduction in infectivity [86], whereas in this study, an approximately 1000-fold reduction was observed for influenza A at 10.4 kGy. The different experimental setups in the two studies may account for the difference in inactivation rates. However, this result also suggests that the effects of electrons on viral activity are similar at comparable doses, regardless of whether high- or low-energy electrons are used. In contrast to influenza A (H3N8), PRRSV, the second single-stranded RNA virus analyzed in this study, was completely inactivated with a dose of 10.4 kGy at similar titers. These differences could be due to differential sensitivity to irradiation caused, for example, by different genome structures (one RNA strand in PRRSV versus a segmented genome in influenza A) and different bacterial genera. These results have opened new avenues for the development and production of inactivated vaccines with improved efficacy [26].

#### X-rays generated by electron accelerators

X-ray as an ionizing radiation can be used in a variety of downstream detection and functional assays because it preserves important biochemical and immunological properties. Industrial X-rays are generated by electron beam accelerators coupled with a metal target (such as tungsten) and used for antimicrobial. Commercially, the accelerated electrons can be converted into X-rays with a continuous spectrum of energies up to 6.2 MeV via a tungsten target [87].

It has also been reported that electrons can produce much less Bremsstrahlung than secondary photon radiation when electron beams (such as LEEI) are used to inactivate pathogens in liquids. In German, "Bremsstrahlung" means the radiation emitted when electrons are decelerated when bombarding a metal target [88]. X-rays originate from atomic electrons and from free electrons that are decelerated near atoms (i.e., Bremsstrahlung). Bremsstrahlung produces a continuous X-ray spectrum, while characteristic X-rays are produced in specific narrow energy bands [88].

X-ray sterilization is still relatively new in terms of understanding the changes that might occur in product materials and functionality compared to the changes observed following gamma radiation processing. The gamma and X-rays are comparable in terms of microbicidal inactivation, predicting a major advantage to switching the industrial sterilization process from gamma rays to X-rays [89]. The technical advantages of this change include increased sustainability of the sterilization supply, better dose uniformity and less oxidative stress when X-rays are used to disinfect certain materials [90]. Furthermore, X-ray sources do not suffer from radio-isotopic half-life decay or the same security implications that are associated with the use of ‘live’ radiological sources. Energy-spectra and dose rates are also more adjustable with X-rays than with gamma sources [91]. On the other hand, applying different radiation modalities with the same range of radiation energies does not show significant differences in the rate of microbial lethality. Radiation resistance of pathogens may not be affected by the energy level of the radiation source and the rate of the dose delivered. In conclusion, the sterilization doses can be safely transferred between modes and sources of irradiation. As long as there is evidence that the specified dose is delivered, dose equals dose, regardless of the type of irradiation source (gamma, X-ray or electron beams) [92].

The production of non-infectious material containing whole-virus characteristics is a distinct advantage. The virus inactivation using X-rays can retain biological and immunological characteristics, as it has been proven accurately using Monte Carlo modeling and the 1-log10 decimal reduction value (D-value) prediction [27, 93]. Afrough et al. provided D-values to achieve controlled virus inactivation and defined the amount and type of X-ray radiation required to produce replication deficient of RNA viruses including Zika virus, Hazara virus, Bebaru virus and Rift Valley Fever virus. By extending this study, the Monte Carlo radiation transport code MCNP6 has been used to model dosimetry for biological pathogens including viruses placed within a MultiRad 225 irradiation chamber. Characterizations of the photon fluence-energy distributions generated by the X-ray tube were achieved for 220 and 190 kV beam potentials, with and without aluminium and copper beam filters of different thicknesses [93]. The results provide evidence that X-ray irradiation can be a tool for rapidly developing reagents for a range of downstream applications requiring high-fidelity nucleic acid or protein substrates [27].

## Non-ionizing radiation

### Blue light

Blue light is considered to be the high-energy visible (HEV) in the 380 to 500 nm range. Blue light is sometimes further broken down into blue-violet light, which is so-called harmful blue light, (approximately 380 to 450 nm) and blue-turquoise light (roughly 450 to 500 nm). Like ultraviolet radiation, high-energy visible blue light has both benefits and risks. There are some concern about long-term effects of blue light from digital devices on the health of our eyes, although more research is needed to determine how much blue light radiation from sunlight and digital devices is “harmful blue light” to the retina [94]. Vatter et al. studied the effect of visible violet-blue lights (405 nm) [29] and (455 nm) [28] on the bacteriophage phi6, as a surrogate of SARS-CoV-2. First, they showed that 4.5 h irradiation of violet-blue light (405 nm) with a dose of 1300 J/cm^2^ reduces the virus PFU concentration by three log-levels [29]. Subsequently, they extended this study towards higher wavelength of blue light (455), and observed the same virucidal effect with radiation dose up to 7200 J/cm^2^ over a period of up to 40 h [28]. In similar studies, Rathnasinghe et al. [30], demonstrated that irradiation with low intensity (0.035 mW/cm^2^) blue light (405 nm) yielded a reduction of log10 0.3288 inactivation after 4 h (0.5 J/cm^2^) and a total log10 1.0325 inactivation of SARS-CoV-2 after 24 h (3.02 J/cm^2^). In further optimization studies, they concluded a both time and dose dependent inactivation of infectious viruses by blue light (405 nm). They also performed similar studies with encephalomyocarditis virus (EMCV) and influenza A virus (IAV), which served as models for non-enveloped and enveloped viruses, respectively. This comparative study indicates that non-enveloped viruses may require higher doses of visible light.

These intriguing studies may open a new window on the application of visible light for antiviral purposes. However, further studies should be conducted to first prove the accuracy of the data and decipher the mechanism. In future designs, the light source should emit a narrow peak of defined wavelength (405 nm) to avoid the unwanted presence of UVA (390 nm) within the source. Theoretically, the ionizing radiation of UVA (390 nm) is a source of oxidative stress upon viral capsids, in compared to blue light (> 405 nm) which does not have sufficient photon energy to release a bound electron from a single atom or molecule, therefore it is not considered as ionizing radiation. In this situation, increasing the emitting wavelength from 405 nm towards 450 nm will increase the accuracy of light source. Further molecular studies will reveal the anti-viral mechanism of blue light via possibly viral genome damage, comparing with UV which can lead to signature mutations and genome damage.

The exact anti-viral mechanisms of blue light are still unclear; however, the role of reactive oxygen species (ROS) is still controversial and under investigation. Studies on bacteria and fungi (reviewed here [95]) demonstrate that the mechanism is associated with absorption of blue light via endogenous microbial intracellular light receptors (chromophores), such as porphyrin photosensitizers and flavins which results in the release of ROS. Despite the virions do not contain endogenous photosensitizers, it has been shown the increased susceptibility of viruses to blue mediated inactivation in the absence of exogenous photosensitizers [30, 96]. Furthermore, blue light-mediated virus inactivation could be improved in the presence of exogenous photosensitizers or in body fluids, e.g., saliva, feces, and blood plasma [96]. The latter implies that the long-term use of blue light to inactivate viruses in blood is unsafe for clinical use because the undesirable ROS may damage not only the virus in blood plasma but also the healthy cells.

In contrast, the use of blue light for the treatment of viral infectious diseases is a new area that warrants further investigation. However, the blue light-based therapy is limited to exposed tissues, because the penetration depth of blue light into tissue is lower than that of red light. Furthermore, blue light technology could be developed for the decontamination of air, surfaces and equipment in hospitals, as well as in other indoor locations, where transmission of viral pathogens is a significant occurrence. As an example, Norovirus (NoV) is environmentally stable and resistant to disinfection, causing gastroenteritis outbreaks often in the hospitals. Blue light could be used as a safe decontamination technology to overcome the NoV outbreaks in hospitals [96].

Any kind of blue light decontamination technology should be effective and safe with commercially impractical irradiance levels. It is important to decipher the photosensitizer-independent mechanism of blue light, to find the safest and most effective wavelength and power density. Finding a specific wavelength of visible light for virus inactivation could be a turning point because of its advantages over UVC. To understand the mechanism, some advances in real-time super-resolution microscopy may visualize the physical changes of viral envelope and genome during exposure to blue light [97]. Moreover, future studies should focus on virions with different physical features regarding the size, shape and compositions (enveloped and non-enveloped). The virion’s size may play a role in photoelectric absorption, while the envelope may have a potential role for a mediated reaction during deformation process.

### Photo-biomodulation (PBM)

Photobiomodulation (PBM) employs low-level non-ionizing forms of blue, green, red or near-infrared (NIR) light. PBM, also known as Low Level Laser Light therapy (LLLT), is a photon therapy based on the use of a light source, such as lasers and light-emitting diodes (LEDs). While PBM with red light can reduce inflammation and stimulate collagen production, PBM with blue light can destroy acne-causing bacteria and other pathogens. PBM includes intravenous laser therapy which works directly into the bloodstream, and transcutaneous laser therapy, which targets deeper tissues, such as the transcranial brain. The effects of PBM appear to be in the range of visible light and NIR, however, more current investigations have identified distinct optical windows within the NIR spectrum (810 nm and 1064 nm) with marked differences in the production of oxygenated hemoglobin and cytochrome c oxidase [98]. PBM laser therapy has various effects on cell proliferation and activation of cell signaling cascades, including the release of ROS and nitric oxide (NO). Since PBM may induce cell proliferation of both normal and malignant cells caution should be exercised and further studies performed to establish the safety of PBM for use against viral-infected cells. This mechanism does not imply for virions due to the lack of ROS generation [99].

LLLT has been widely used to treat viral infections and involves the exposure of cells or tissues to low levels of red and NIR. Lugongolo et al. [34, 100] investigated the inactivation of HIV-1 infected and uninfected TZM-bl cells using LLLT alone [34] or combined [100] with anti-viral efavirenz, an HIV reverse transcriptase inhibitor. In the model of LLLT alone [34], the infected and uninfected cells were irradiated at a wavelength of 660 nm with fluencies of 2–10 J/cm^2^. The results showed that infected cells irradiated at 2, 4, 6, and 8 J/cm^2^ showed no changes in cellular viability, whereas infected cells irradiated at 10 J/cm^2^ showed a significant decrease in viability compared with the control. The results showed no statistically significant differences between uninfected and infected cells, except for infected cells irradiated with 10 J/cm^2^. The significant decrease in luciferase luminescence in irradiated cells suggests that laser irradiation impairs HIV infection. In the second model, the combination of LLLT and efavirenz significantly reduced HIV infection in cells, despite the undesirable effects observed in the cells as demonstrated by cell morphology, proliferation and cell integrity assay [100].

In another study, Liu et al. investigated the membrane receptor-mediated signal transduction mechanism (STM) leading to PBM. They used the light spectrum from UVA to IR to inactivate SARS virus. The reason for this phenomenon is the conformational changes of envelope (Env) glycoproteins, which are sensed by the light-induced electron excitation, while the frequency of light absorption of Env glycoproteins increases from UVA (320–400 nm) to IR. According to many studies, this light absorption is non-resonant and its transition rate is very insignificant, which can be enhanced by a similar and independent Env glycoprotein [19, 49, 101].

### Ultra-short pulse (USP)

Ultrashort pulse lasers (USPLs) emitting visible to near-infrared light have been used to inactivate a broad spectrum of viruses. One of the promising candidates for virus inactivation is the use of femtosecond lasers (fs) in the visible and near-infrared range. Tsen et al., [102–106] evaluated ultrashort pulsed lasers and their application for pathogen inactivation. They also investigated low-power ultrashort pulsed (USP) lasers operating at 425 nm and in the near-infrared region, and documented that these wavelengths can inactivate viruses, such as murine cytomegalovirus (MCMV), M13 bacteriophage, and HIV [102]. In addition, some viruses, such as the non-enveloped Murine norovirus virus 1 (MNV-1) and the M13 bacteriophage, could be inactivated with a USP laser by a physical mechanism called Impulsive Stimulated Raman Scattering (ISRS) [102]. The ISRC process makes the USP laser treatment an attractive potential method against viral infection [104, 107] and also enables the production of safe, effective, chemical-free fully inactivated viral vaccines against influenza [108].

**Fig. 3:**
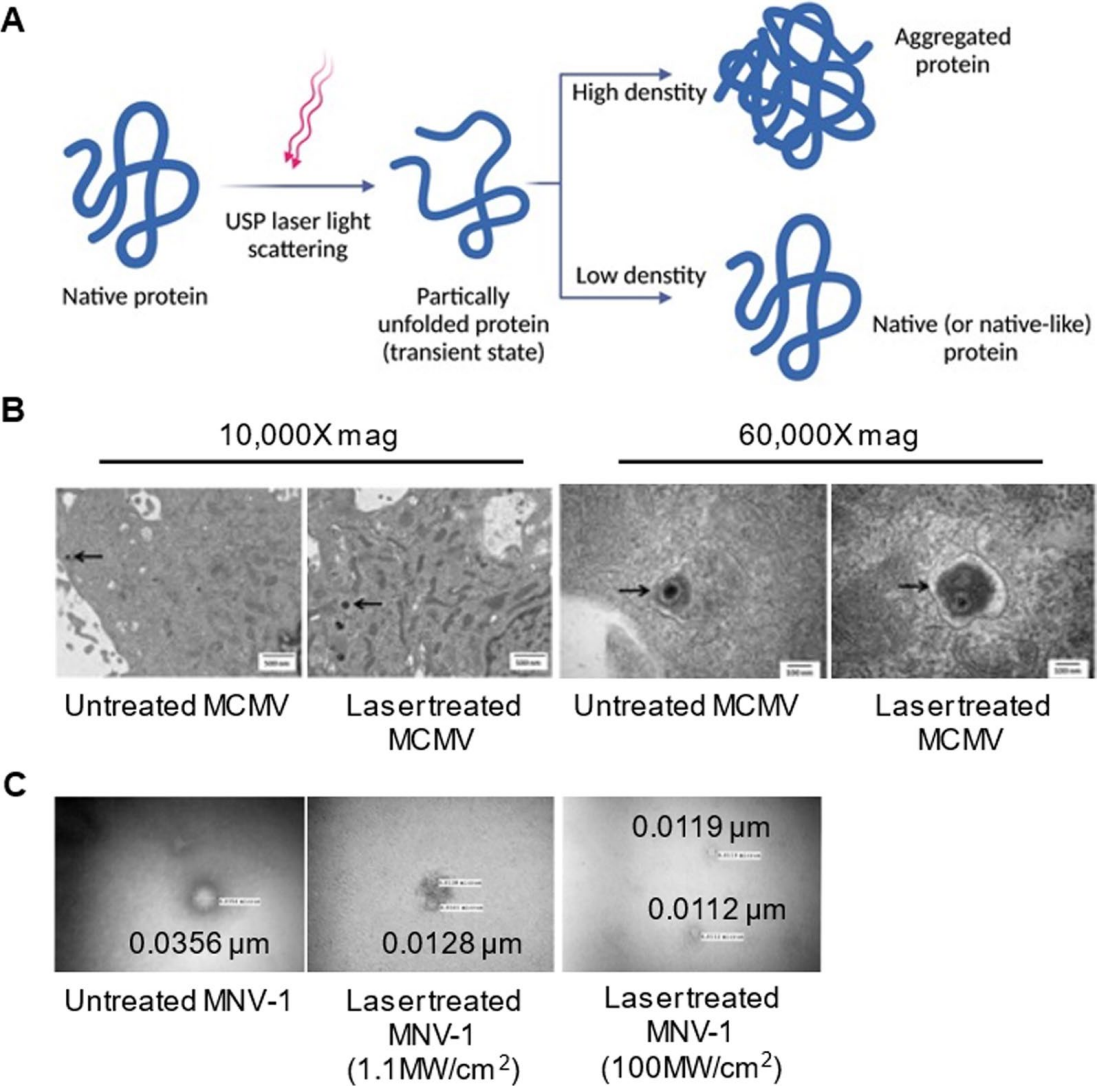
**A** Proposed model for ultra-short pulsed laser-induced protein aggregation. One of the promising candidates for virus inactivation is using femtosecond (fs) pulse lasers in visible and near-infrared ranges. **B** Electron microscopy observation of cells infected by murine cytomegalovirus (MCMV). The laser-treated virion can enter into host cells, but with no viral replication or gene expression due to genome damage. Adapted and reprinted from [31], Copyright 2014, with permission from Elsevier

Another concept the researchers investigated was the dependence of USP laser irradiation on virus internalization, replication, or gene expression in cells [107]. The continuous sequence of 60 fs pulses is delivered by a laser with a repetition rate of about 80 MHz. The output of the second harmonic generation system of the Ti–sapphire laser was used to stimulate the samples (Fig. 3A). For this purpose, Murine Cytomegalovirus (MCMV), an enveloped DNA virus, was studied as a virus model [31]. By using electron microscopy and fluorescence microscopy to detect intracellular virions, they found that MCMV virions treated with laser were successfully internalized into host cells, which was confirmed by detection of intracellular viral DNA by PCR (Fig. 3B). The observations indicated that after laser treatment, the viral DNA itself remained amplifiable by polymerase, but viral replication or gene expression was inhibited in cells infected with the laser-treated virus [31]. These results, which are consistent with previous studies [102, 103, 109], demonstrate that laser treatment stabilizes the capsid, thereby arresting the decapsulation of the capsid in the cells. Interestingly, the mechanical properties of viral capsids can be targeted by ultrashort pulsed lasers to overcome viral mutational escape and target drug-resistant pathogens.

Studies on the selective inactivation of viruses using NIR sub-picosecond laser show promising results for the disinfection of viral pathogens in blood products and open novel approach for the treatment of blood-borne viral diseases in the clinic. It has been shown that both wild-type and mutant viral strains can be inactivated after a one-minute irradiation with a NIR sub-picosecond fiber laser with a pulse intensity of > 100 GWcm^−2^ [104]. The researchers demonstrated the potential ability of nanopore technology to accurately characterize individual viruses and examined how viral functions are affected by different treatment approaches. They also quantified the efficacy of this label-free virus inactivation technique [110].

Berchtikou et al. [32] utilized the high-energy fs laser treatment to sufficiently inactivate M13 bacteriophages, which served as surrogates, for studying the virus inactivation methods. They used fs lasers with a large volume (1 cm^3^), short exposure time (few minutes), and. They found that a pulse width of 40 fs and a power density of 0.6 TW.cm^−2^ resulted in minimal DNA damage (4 ± 2% at 800 nm and 5 ± 2% at 400 nm). Virus inactivation was significantly enhanced by increasing the density of laser energy and its pulse width. Moreover, they observed a load reduction at 2.73 ± 0.14 on the log10 scale of the M13 bacteriophage viability after 2 min of the laser irradiation with a 40-fs pulse at 400 nm, whereas excitation with a 40-fs pulse at 800 nm only led to a load reduction of 1.38 ± 0.13 on the log10 scale of viability after 8 min irradiation. With simultaneous irradiation at both wavelengths, they observed a moderate response. Moreover, the second harmonics of Ti:sapphire showed higher inactivation efficiency at short exposure times compared to the fundamental wavelengths [32]. For high density proteins, accumulation can occur between mere hydrophobic fragments on adjacent proteins. In contrast, low-density proteins quickly reassume their original conformation. The packed spheres represent the hydrophilic regions of the protein, while the unfilled spheres represent the hydrophobic regions [31].

The ability of the USP laser for viral inactivation could be improved using singlet oxygen enhancers. Kingsley et al. [33], assessed the sensitivity of MNV (Murine Norovirus) to USP laser in the presence of singlet oxygen. For this purpose, they employed a tunable mode-locked Ti–Sapphire laser at 425 nm. These pulses irradiated a doubled frequency to make fs pulses at wavelengths 400, 408, 425, 450, 465, and 510 nm with an average power of 150 mW. In this study, inactivation of MNV by singlet oxygen was evaluated by using singlet oxygen enhancers (Rose bengal and riboflavin and methylene blue at a final concentration of 0.1%). The effects of viral inactivation compared with untreated controls was assessed by using oxygen quenchers. The results suggested that the mechanism of the laser induced inactivation of viruses is independent of wavelength. Also they used a continuous wave (CW) wavelength (408 nm) and observed that this type of laser could significantly inactivate MNV during irradiation. The observations indicated that singlet oxygen generation using photosensitizers substantially improves the efficiency of the inactivation. The study concluded that using singlet oxygen enhancers USP is not necessary for virus inactivation.

**Fig. 4:**
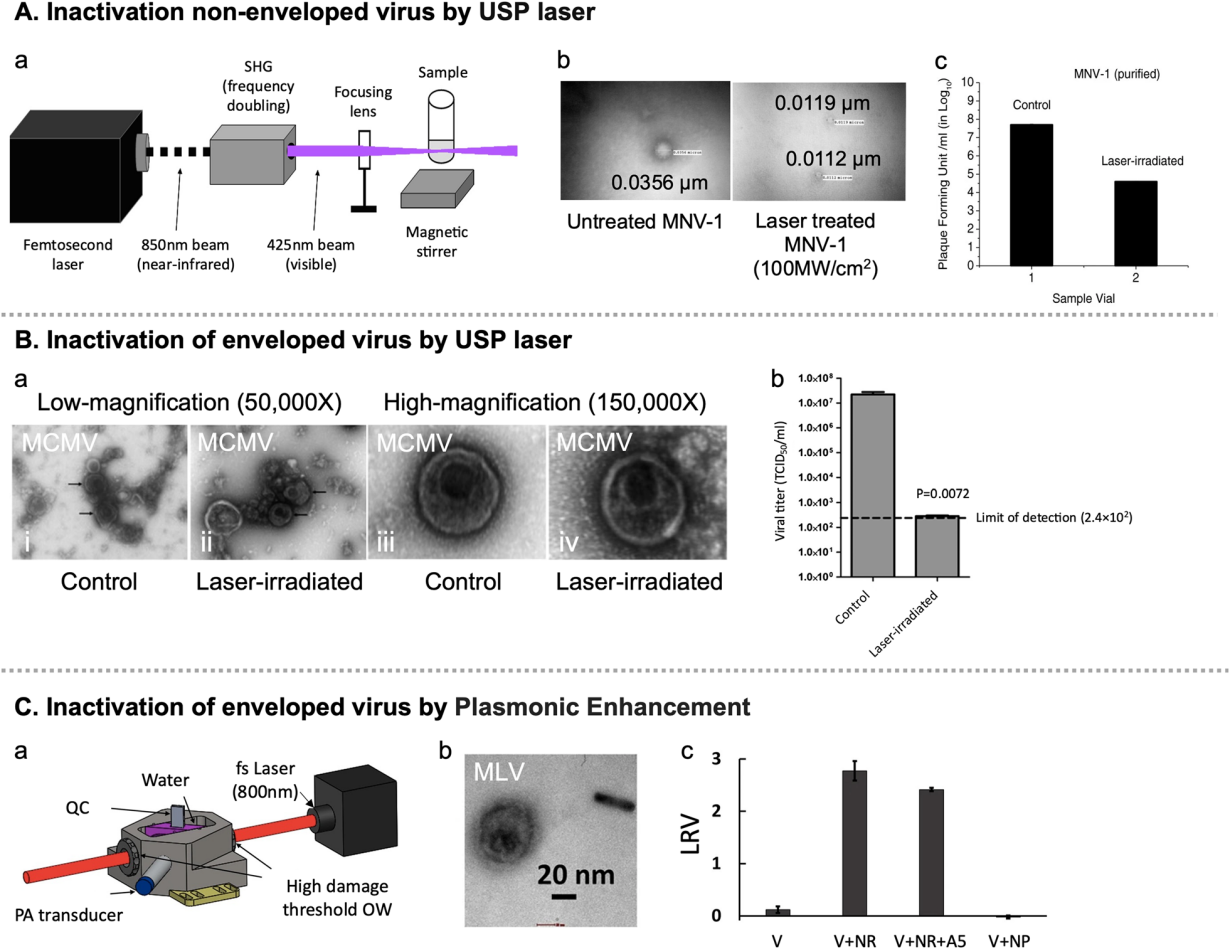
Three models of viral infection using USP and plasmonic enhancement depicted instrumental mechanism and results. **A**, **B** Experimental setup for femtosecond laser irradiation using NIR laser beam at 850 nm which was frequency doubled to generate 425 nm blue light. This instrument used for both non-enveloped MNV-1 [103] (**A**) and enveloped MCMV [109] (**B**) virions. **C**, **a** Schematic of experimental setup for photoacoustic measurements. The following abbreviations are used: fs: femtosecond, QC: quartz cuvette, PA: photoacoustic, and OW: optical window. **C**, **b** TEM image of laser irradiated Murine Leukemia Virus + PEGylated nanorod. **C**, **c** Virus log-reduction-value (LRV) measured for virus (V) alone, virus + PEGylated nanorods (NR), virus + annexinV functionalized nanorod (NR + A5) and virus + spherical nanoparticles (NP) after irradiation to a pulsed (35 fs) 805 nm laser with an average power of 3 W for 10 s exposure time [113]

By correlating viral inactivation with the structural and molecular effects, mechanisms for the inactivation of enveloped and non-enveloped viruses by fs laser irradiation were deciphered. The anti-viral effect of USP laser at low mean irradiance is employed via impulsive stimulated Raman scattering, whereby high-frequency resonance vibrations provoke sufficient mechanical vibrations to break non-covalent bonds and subsequently disintegrating into protein subunits on capsid of non-enveloped viruses (Fig. 4A) [103].

In contrast, USP laser irradiation promotes vibrations to the capsid proteins of the enveloped viruses, but without breaking/dissociating the capsid. Since the concentration of confined proteins is remarkably high within the capsid of a virion, causing cross-linkage with other neighboring proteins, leading to the protein aggregation (Fig. 4B) [109]. An intense laser pulse could generate shock wave-like vibrations upon impact with the virus, promoting viral inactivation [103]. Simulations of the M13 bacteriophage capsid revealed that a high laser intensity of USP is required for effective ISRS [111]. In opposition to aforementioned studies on M13 bacteriophage inactivation, Wigle et al. [112] reached to contradicting results and was unable to reproduce inactivation of M13 bacteriophage through fs laser irradiation highlighting the need for further research on the mechanism of inactivation.

There are some alternative mechanisms to effect structural and chemical transformations, including multiphoton absorption. As an alternative strategy to overcome some of the challenges associated with pulsed laser driven virus inactivation, Nazari et al. [113] investigated the plasmonic enhancement of photonic inactivation of Murine Leukemia Virus (MLV) via 805 nm femtosecond pulses through gold nanorods. A plasmonically enhanced inactivation of MLV was observed in as little as 10 s of irradiation with incident laser powers ≥ 0.3 W, resulted in ≥ 3.7-log reduction. Interestingly, no damage to co-incubated antibodies was observed, signifying the selective virus inactivation (Fig. 4C).

#### USP to promote transferring anti-retroviral drugs

Malabi et al. [35] used the pulsed fs laser (800 nm) with an optimal power of 4 μW and a cell exposure time of 10 ms to deliver different amounts of anti-retroviral drugs (ARVs) including Nevirapine, Efavirenz, Ritonavir, and Raltegravir into HIV-1 infected TZMbl cells. Then, alterations in cellular responses were assessed in cellular morphology, viability, cytotoxicity, and luciferase activity. In the presence of ARVs, laser-exposed cells showed a significant reduction in viral infection and cell viability and increased cytotoxicity. This study proved that fs laser pulses effectively promote the transmission of ARVs to HIV-1-infected TZMbl cells and lead to a significant reduction in HIV-1 infection. Maphanga et al. [114] used fs laser pulses for the targeted in vitro delivery of antiretroviral (ARV) drugs into HIV-1 infected cells. In the experiment, HEK 293 T cells were used to produce HIV-1 enveloped pseudovirus (ZM53) to infect TZM-bl cells, which were later treated with laser pulses emitted by a titanium sapphire laser (800 nm, 1 kHz, 113 fs, ~ 6.5 µW) to create sub-microscopic pores in the cell membrane to allow the influx of extracellular medium. Therefore, optical delivery of ARV’s increased drug absorption, enhanced drug efficacy, subsequently reduced HIV-1 infection.

## Approaches of selective virus inactivation using light

Viruses are unlikely to increase resistance to light radiation, as is the case with drugs. However, ionizing radiation affects different tissues regardless of their location in the body and does not have the complicated delivery to different body compartments. Thus, an alternative strategy is to eradicate viruses from infected reservoirs using targeting-based approaches. Attaching markers such as fluorescent dyes to viruses using different strategies and the resulting generation of individual emission wavelengths could enhance the ability and selectivity of lasers to inactivate viruses. The use of light beams as inactivators and exciters for viral genetic content or receptors can be combined with photosensitizers to improve damaging the viral membrane. The selectivity of this photo-inactivation strategy could be improved via conjugation with antibodies targeting either virus envelope [115] or envelope-expressing infected cells [49]. However, the ideal treatment should use light and no mediators to minimize side effects and, of course, costs. With this viewpoint, an attractive strategy for selective inactivation of viruses is the use of NIR sub-picosecond laser [113] and visible ultrashort pulsed laser [103, 109] which have showed promising results for treatment of blood-borne viral diseases.

Viral inactivation of tissues and organs could be accomplished by low energy lasers or fs lasers. It is believed that injection or inhalation of nontoxic nebulized photosensitizers into the lungs and the delivery of appropriate light irradiation into deep organs may be practical to kill respiratory viruses such as Influenza [116]. Another alternative to virus inactivation in infected organs could be an innovative combination of normothermic ex vivo perfusion and light-based inactivation using infected human lungs. This strategy has been used to inactivate HCV virus in HCV-infected human lungs in a short period of time during lung transplantation [117].

## Conclusion and future perspective

We review here the rapid advances in the use of physical irradiation methods for viral inactivation. Compared to chemical drugs and materials used to inactivate viruses and treat diseases, the variety of physical techniques that include electromagnetic light waves with wavelengths of gamma rays, X-rays, UV rays, and visible light offer unique opportunities for eliminating deadly viruses. In our review, for each innovative method we decipherer the mechanisms, discuss the key parameters and showcase the recent validation experiments towards efficient viral inactivation, already lead to a range of new tools and devices developed with improved performance. Through detailed analysis, we show that new advances in the characterization of viral genome and proteins during irradiation will be the key to better understanding of mechanism and quantifying each key parameter for efficient viral inactivation without harmful side effects to human and the environment.

Besides, studies on the viral-cell entry during irradiation are also important to develop viable approaches of viral inactivation in vaccine production, downstream diagnostics, and directly in the body for treatment of patients. For enveloped viruses, the irradiation strategies causing the damage of viral envelope proteins are advantageous over the other strategies destroying nucleic acids, to prevent virus mutations. When choosing the appropriate method, we should consider size, shape, structure, and type of the virus. When it goes to antiviral therapy, the implementation ability, repeatability, affordability, and lack of long-term toxicity of the method are the most important factors.

The use of low-energy lights to generate shock wave-like mechanical vibrations can be promising, due to the minimal damage to tissues, high potential for damaging various parts of the virus, reduced treatment time, and deep penetration of the virus into the tissues. The mechanical vibrations on the virus structure could be provoked using pulsed laser via impulsive stimulated Raman scattering, or plasmonic enhancement, while future advances in resonance vibrational studies may improve light-based virus inactivation.

Our world is facing many threats: COVID -19, the many potential viral epidemics and possible future pandemics that have already caused many deaths in all countries facing limited medical resources. Therapies using physical waves and in combination with chemical drugs and materials may produce synergistic effect for virus inactivation. Irradiation strategies are preferred because they do not destroy the cells and our bodies, are environmentally friendly, and are cost-effective. However, compared to thermal methods and ionizing approaches, it is important to ensure high efficiency of virus inactivation due to the mild nature of non-ionizing strategies. Therefore, further research is needed to fully understand the potential for viral effects, cytotoxicity, and possible viral recovery after non-thermal methods.

## Outlook

- Using physical waves and irradiations with non-destructive controlled parameters are promising to inactivate viruses, including SARS-CoV-2, without the risk of drug resistance.
- Recent reports from different research groups claim the non-ionizing blue light can inactivate the virus in an unknown mechanism. These promising results highlight the use of visible light technology for the application of continuous decontamination in occupied areas such as hospitals.
- Recent studies aim to develop novel photonic inactivation approach that is selective to pathogens, with neither compromising the protein-based pharmaceuticals, nor specific targeting to the pathogens.
- The emerging fields of physical irradiation with different mechanisms (e.g., ionizing LEEI with non-ionizing PBM) will enhance the existing methods for optimized viral inactivation towards enhanced efficacy, long-term use, and ideally high selectivity.
- The advances in the selective inactivation of viruses using NIR sub-picosecond laser and visible ultrashort pulsed laser open novel approach for the treatment of blood-borne viral diseases

## Abbreviations

D-Value: 1-Log10 decimal reduction value
ARV: Antiretroviral
EMCV: Encephalomyocarditis virus
Fs: Femtosecond lasers
HEV: High-energy visible
HIV: Human Immunodeficiency Virus
IAV: Influenza A virus
LEDs: Light-emitting diodes
LLLT: Low Level Laser Light Therapy
LEEI: Low-energy electron irradiation
MERS-CoV: Middle East Respiratory Syndrome Coronavirus
MDP: Mn-Decapeptide
MLV: Murine Leukemia Virus
MNV-1: Murine Norovirus 1
NIR: Near-infrared
PPE: Personal protective equipment
PBM: Photo-biomodulation
PFU: Plaque forming unit
ROS: Reactive oxygen species
TCID50: Tissue culture infectious dose
USP: Ultra-short pulse
